# Impact of COVID-19 Pandemic on the Italian Humanitarian Congenital Cardiac Surgery Activity: What No One Tells You

**DOI:** 10.3389/fcvm.2021.705029

**Published:** 2021-07-28

**Authors:** Alessandro Giamberti, Federica Caldaroni, Alessandro Varrica, Carlo Pace Napoleone, Stefano Maria Marianeschi, Nicola Uricchio, Vittorio Vanini, Francesco Santoro, Giovanni Battista Luciani, Giovanni Stellin, Gaetano Gargiulo, Bruno Murzi, Sergio Filippelli, Guido Oppido, Salvatore Agati, Lorenzo Galletti, Alessandro Frigiola

**Affiliations:** ^1^Department of Congenital Cardiac Surgery, IRCCS Policlinico San Donato, San Donato Milanese, Italy; ^2^Association “Bambini cardiopatici nel Mondo” Non-Governmental Organization (NGO), Milan, Italy; ^3^Pediatric Cardiac Surgery, Regina Margherita Children's Hospital, Torino, Italy; ^4^Pediatric Cardiac Surgery Unit, Grande Ospedale Metropolitano Niguarda, Milan, Italy; ^5^Cardiac Surgery, Aziende Socio Sanitarie Territoriali (ASST) Papa Giovanni XXIII, Bergamo, Italy; ^6^Heart of Children Foundation, Bergamo, Italy; ^7^Missioni Cardio Chirurgiche Internazionali, Gaslini Pediatric Hospital, Genova, Italy; ^8^Pediatric and Congenital Cardiac Surgery Unit, Department of Surgery, Dentistry, Pediatrics, and Gynecology, University of Verona, Verona, Italy; ^9^Pediatric and Congenital Cardiac Surgery Unit, Department of Cardiac, Thoracic and Vascular Sciences and Public Health, University of Padova, Padua, Italy; ^10^Pediatric and Grown-up Congenital Cardiac Surgery, University of Bologna, S. Orsola-Malpighi Hospital, Bologna, Italy; ^11^Pediatric Cardiac Surgery Unit, Heart Hospital, G. Monasterio Foundation, Massa, Italy; ^12^Department of Pediatric Cardiac Surgery, Bambino Gesù Children's Hospital, Rome, Italy; ^13^Congenital Cardiac Surgery Unit, Monaldi Hospital, Naples, Italy; ^14^Centro Cardiologico Pediatrico del Mediterraneo - Bambino Gesù, “San Vincenzo” Hospital, Taormina, Italy; ^15^Department of Pediatric Cardiac Surgery, Bambino Gesù Children's Hospital IRCCS, Rome, Italy

**Keywords:** congenital heart diasease, cardiac surgery, health, COVID-19, humanitarian medicine, developing countries, cooperation

## Abstract

More than 4 millions of children with congenital heart disease (CHD) are waiting for cardiac surgery around the world. Few of these patients are treated only thanks to the support of many non-governmental organizations (NGOs). Starting in December 2019, the so-called coronavirus disease 2019 (COVID-19) has rapidly become a worldwide pandemic and has dramatically impacted on all the international humanitarian activities for congenital heart disease. We analyzed data from all the Italian congenital cardiac surgery centers with the aim to quantify the impact of the pandemic on their charities. Fifteen Italian centers participated in the study and contributed to data collection. We analyzed and compared data regarding humanitarian activities carried out abroad and on site from two periods: year 2019 (pre-COVID-19) and year 2020 (COVID-19 pandemic). In 2019, 53 international missions were carried out by Italian congenital cardiac surgeons, resulting in the treatment of 471 CHD patients. In the same period 11 Italian cardiac centers operated on 251 foreign patients in Italy. In 2020, the pandemic led to a reduction of this activity by 96% for the surgery performed overseas and 86% for the interventions carried out in Italy. In conclusion our study shows the important quantitative impact of the pandemic on the Italian humanitarian cardiac surgical activity overseas and in Italy. This shocking result highlights the failure of the systems adopted so far to solve the problem of CHD in developing countries.

## Introduction

Congenital heart diseases (CHD) occur in 1% of live birth and, according to data from WHO (www.who.org/health-statistics), there are 1.5 million newborns per year affected by CHD and more than 4 million children waiting for cardiac surgery all over the world (1).

There is a dramatic disproportion between medical access and healthcare services within developed and developing countries, with 89% of the world's healthcare resources used for only 7% of the sick people (World Health Statistics 2015). This unequal distribution and access to healthcare facilities is particularly evident in cardiac surgery for CHD.

In the last 30 years, enormous economic efforts and investments in human and technological resources have been made by many cardiovascular non-governmental organizations (NGOs), with the aim of creating stable clinical services for these patients and the project of setting up autonomous cardiac surgical programs in these countries (2).

Despite all the efforts, only a small percentage of patients, and only thanks to the voluntary activity of many NGOs, receive life-saving treatments.

Starting from Wuhan (China) in December 2019, the disease called coronavirus disease 2019 (COVID-19) has rapidly become a worldwide pandemic, with currently 115 million cases globally and over 1.5 million deaths (3).

For the first time since the end of World War II, the health systems of rich countries have been devastated and transformed by the pandemic.

Treatment and management of patients with COVID-19 became a priority. As result, all elective health services were interrupted or postponed, the economies went through a crisis, and the possibility of moving from one country to another was drastically reduced.

Obviously, all the international humanitarian cooperation activities experienced a dramatic reduction. On behalf of the Congenital Domain of the Italian Society of Cardiac Surgery, we aim to quantify the impact of the pandemic on the cardiac charities.

## Materials and Methods

Using a questionnaire, we conducted a descriptive, retrospective, and comparative survey on the impact of the COVID-19 pandemic on the national and international humanitarian activity of all the Italian congenital cardiac surgeons.

In Italy there are 15 Cardiac Surgery centers dedicated to the treatment of CHD. Inside each center there are cardiac surgeons who devote free time, on a voluntary basis, to humanitarian activities of international collaboration. These activities are managed by different NGOs.

There is no national database for cardiac surgery in Italy and even less is known regarding the activity carried out by Italian cardiac surgeons overseas. Data were collected through a written survey sent to each Italian congenital cardiac surgeon in activity (Supplemental Material).

At the same time, we contacted all the NGOs involved to double check the data provided. The list of NGOs is shown in Table 1.

**Table 1:** The list of NGOs involved in Humanitarian congenital cardiac surgery activity.

**Organization**	**Website**	**State**
Mission bambini	https://www.missionbambini.org/	ITA
Palestine Children's Relief Fund	https://www.pcrf.net/	USA
Save the children	https://www.savethechildren.it/	UK
Gift of life	https://www.giftoflife.org/	USA
Associazione bambini cardiopatici nel mondo	https://www.bambinicardiopatici.it/	ITA
Ufficio Missioni Umanitarie Ospedale Pediatrico Bambino Gesù	http://www.ospedalebambinogesu.it/attivita-internazionali	ITA
Mending Kids	https://www.mendingkids.org/	USA
Novick cardiac alliance	https://cardiac-alliance.org/	USA
Chain of hope	https://www.chainofhope.org/	UK
Un Cuore un Mondo	http://www.uncuoreunmondo.org/	ITA
The Heart of Children	https://www.theheartofchildren.org/	ITA

Data from two periods: year 2019 (pre-COVID-19) and year 2020 (COVID-19 pandemic) were analyzed and compared. The survey was divided in two sections, with questions regarding: (1) The humanitarian activity carried out abroad, which included: the countries involved, the number of operated patients and the name of supporting organization (NGO); (2) The humanitarian activity carried out in Italy, including: the number of patients operated on, the country of origin of the patients, and the sponsoring institution.

### Statistical Analysis

Data collection was performed through a survey search realized by written inquiry. Descriptive statistics for categorical variables are reported as frequency and percentage. The results are summarized in tables and/or bar graph.

## Results

Italy, with over 60 million inhabitants, has 15 cardiac surgery units dedicated to CHD. Among these, 4 units (27%) operate in exclusively pediatric hospitals, whereas, 11 units (73%) operate in institutions offering both adult acquired and congenital cardiac surgery services. Ten units are within general hospitals, three belong to university centers and two are in private hospitals accredited with the National Health System.

All the centers (15/15 = 100%) agreed to participate in the survey and contributed to the data collection.

Twenty-one congenital cardiac surgeons from 11 (11/15 = 73%) cardiac surgery units declared to have participated in international humanitarian activities during the analyzed period.

### Humanitarian Surgical Activity Overseas

**Figure 1:**
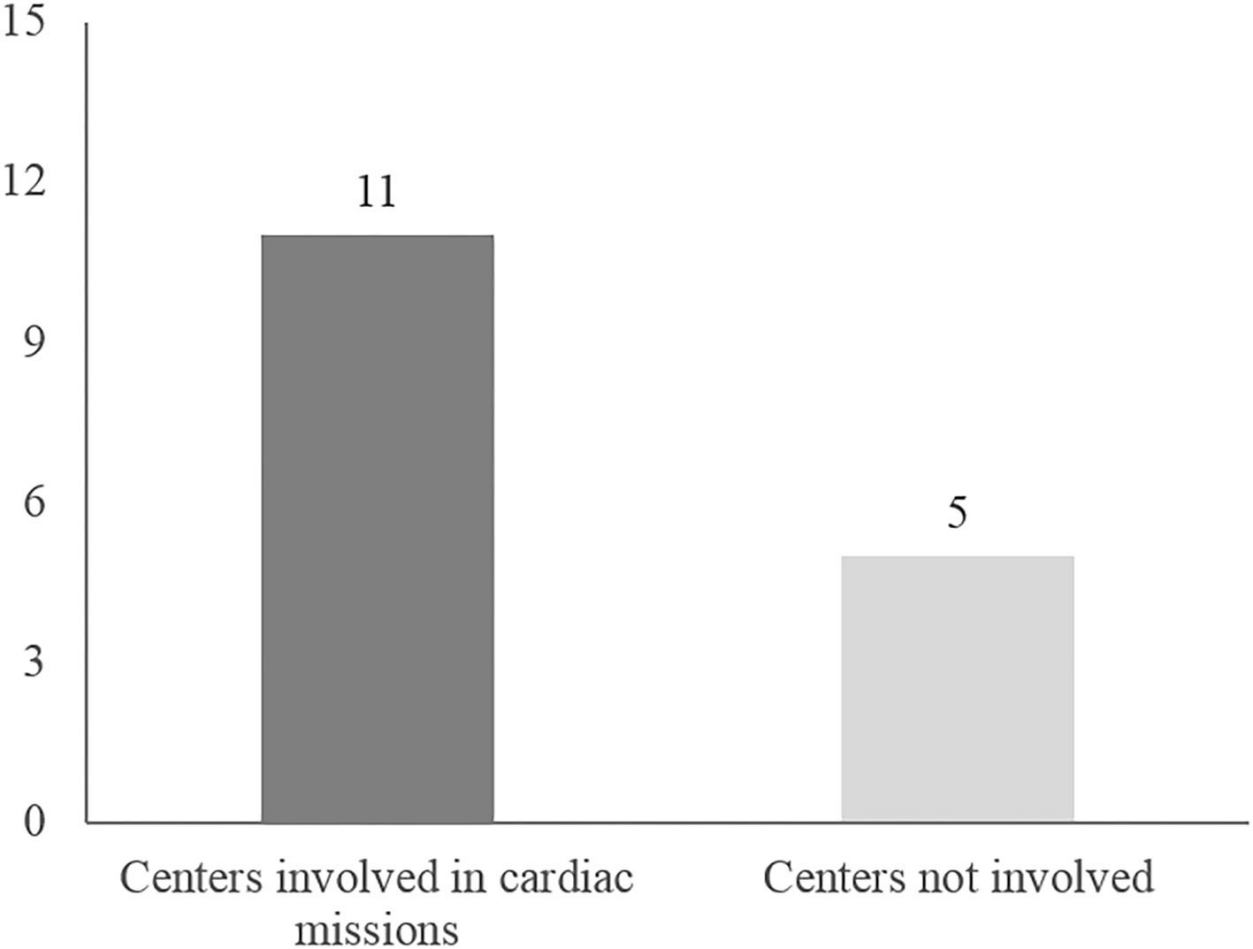
Italian centers involved in pediatric cardiac surgery missions overseas.

**Figure 2:**
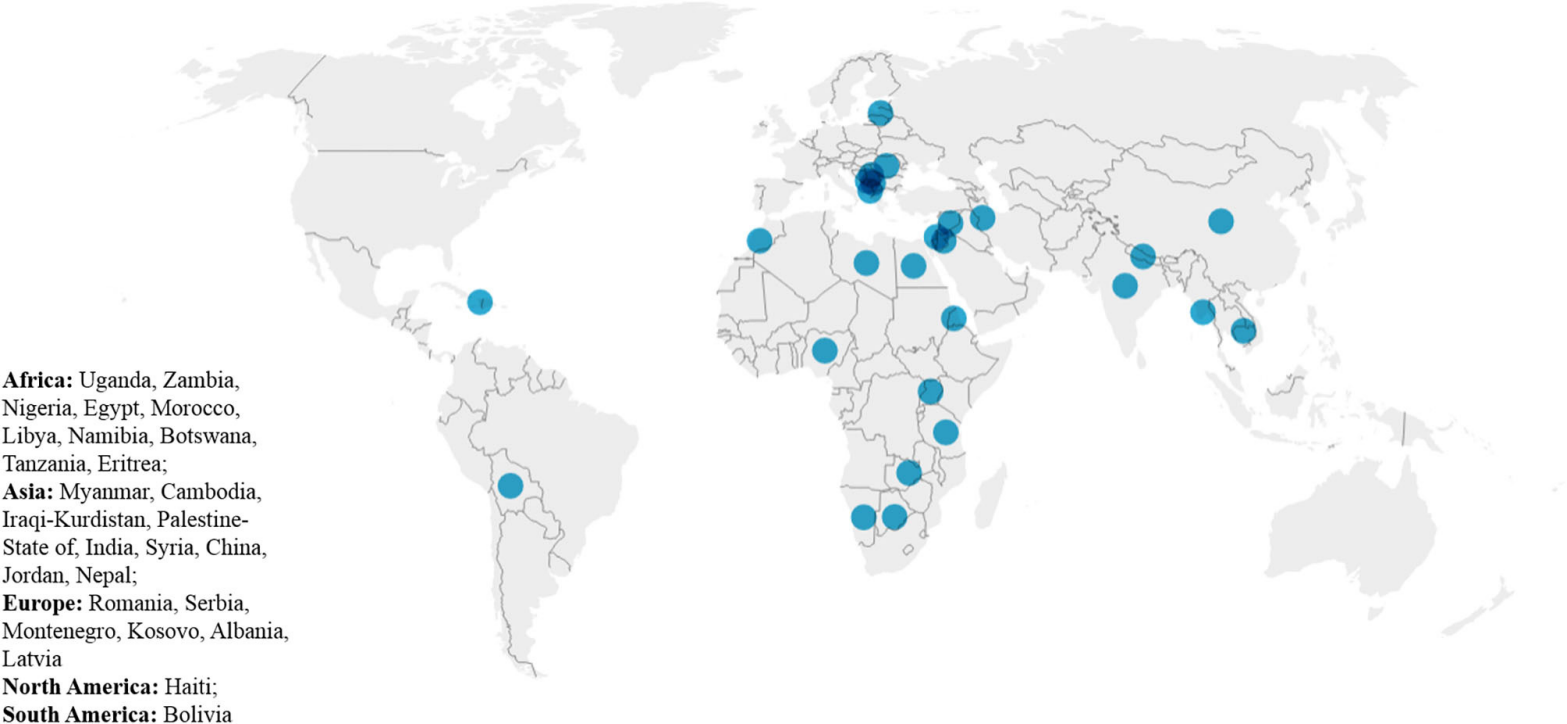
Countries hosting humanitarian surgical cardiac missions. The figure is realized using the data visualization tool Datawrapper.

In the year 2019 twenty-one surgeons from 11 Italian centers have been involved in international humanitarian surgical activity (Figure 1) for a total of 53 surgical missions overseas. Each center performed between 1 and 14 missions per year (Table 2). The list of countries where these charities took place is depicted in Figure 2.

**Table 2:** Missions performed and patients operated overseas by each Italian cardiac surgery centers in 2019 and 2020.

**Italian cardiac** **surgery center** (^*^)	**Missions 2019** **(*n*)**	**Patient treated abroad** **during the missions** **2019** **(*n*)**	**Reduction on** **mission activity** **2020** **(%)**
1	1	15	100
2	4	35	100
3	9	80	100
4	4	50	100
5	14	95	100
6	0	0	0
7	3	22	100
8	1	10	100
9	6	72	100
10	1	15	100
11	0	0	0
12	5	20	100
13	5	57	100
14	0	0	0
15	0	0	0
16	0	0	0

* *Each number identifies a center*.

As a result, 471 CHD patients were operated on (Table 2), with an average of 43 patients (range 10–93) per center and an average of nine patients per mission.

All the activities and patients operated in their developing countries have been sponsored by different Italian or international cardiovascular NGOs and the list is showed in Table 1.

In 2020, the international humanitarian activity carried out by the Italian pediatric cardiac surgery community suffered a dramatic 95% reduction because of the pandemic. Only three missions were performed overseas during the months of January (pre-pandemic) and June-July when it seemed that the pandemic was in a phase of decline.

### Humanitarian Surgical Activity in Italy

In the year 2019, a total of 251 foreign patients were admitted and treated in Italian centers, with an average of 21 patients per center (range 3–130). An exception is represented by a single center with long-standing collaborative relationships with some counties, in which in 2019 a total of 130 patients, mainly from Romania, Tunisia, and Egypt was operated on (Table 3).

**Table 3:** Patients operated by each Italian cardiac surgery centers in Italy in 2019 and 2020.

**Italian cardiac** **surgery center** (^*^)	**Foreign patient treated** **in Italy 2019** **(*n*)**	**Reduction** **in 2020** **(%)**
1	4	100
2	15	100
3	0	0
4	3	100
5	130	90
6	20	60
7	20	80
8	5	75
9	6	50
10	3	100
11	5	100
12	0	0
13	25	0
14	15	100
15	0	0
16	0	0

* *Each number identifies a center*.

**Figure 3:**
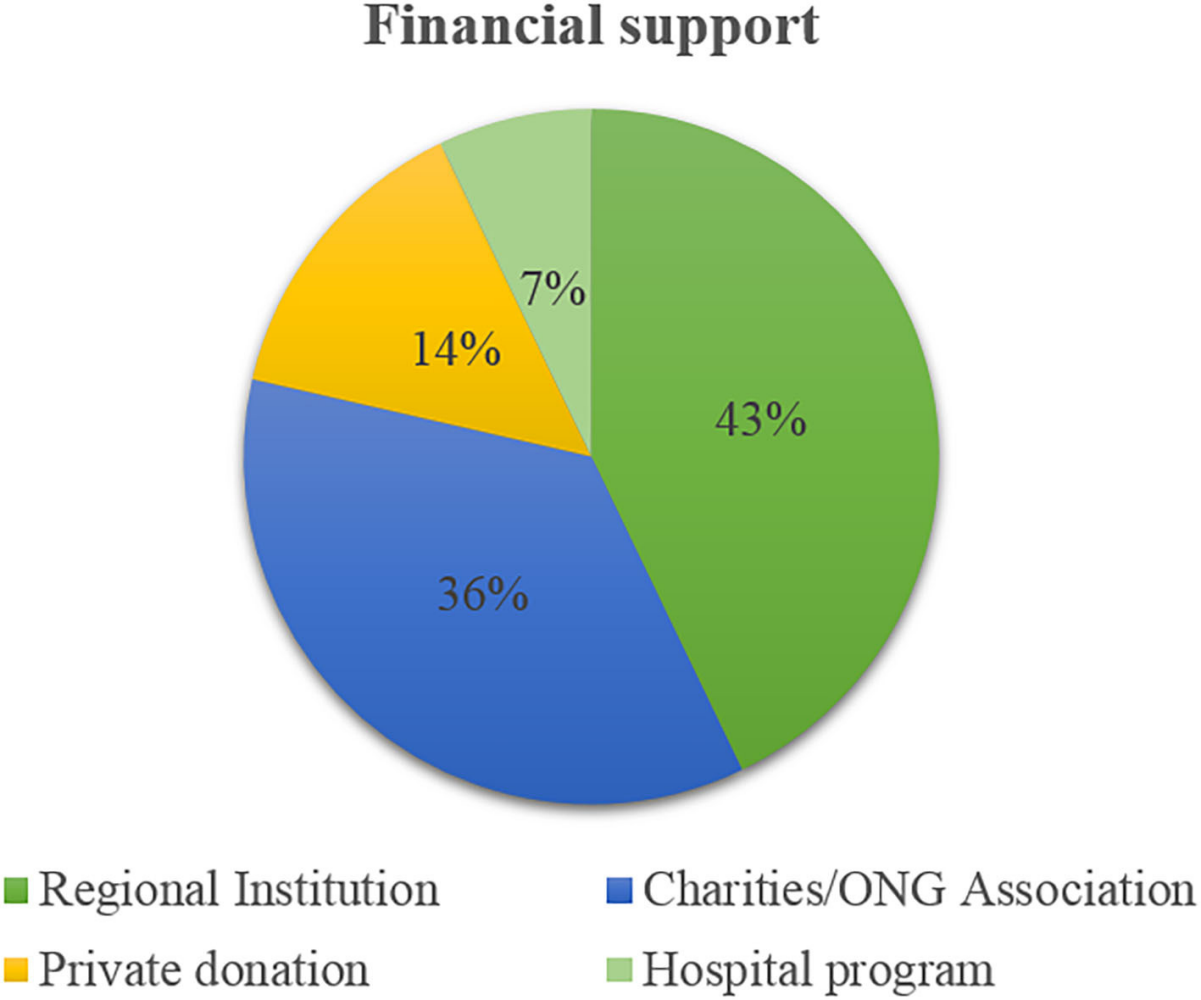
Financial support modalities for patients treated in Italy.

Financial support modalities for patients treated in Italy from overseas is shown in Figure 3.

In 2020, the activity of these charities in Italy experienced a reduction of 86%, with a hypothetical predicted resumption of the activity by the end of 2021 at the earliest, and only after a massive vaccine rollout in the population.

## Discussion

Many advances in pediatric cardiology and cardiac surgery have occurred during the past 40 years and most of the congenital heart disease (CHD) are now antenatally diagnosed and treated in neonatal age, with the result that over 90% of these patients in Western Europe or North America can expect to reach adulthood.

Every year, 1.3 million children are born with a congenital heart disease. However, only <100.000 have access to lifesaving treatments, leaving behind over one million of children every year without any form of medical care (4). The cumulative numbers create a global waiting list for cardiac surgery of more than 4 millions of pediatric patients in the world (5).

Nowadays, the majority of these children (90%) could be treated, saved, and subsequently have a good quality of life. Unfortunately, most of them are destined to die because they live in poor countries with suboptimal care or no access at all to health facilities.

Many efforts have been made by non-governmental organizations (NGO) and private voluntary organizations (1, 2, 6, 7) to reduce this disparity and provide better health coverage, but, to date, the results are minimal, and a great deal still needs to be done.

Currently, there are three different organizational models to establish a cooperation project for pediatric cardiac surgery in developing countries, and all of them have their own limitations.

The first is to send patients needing treatment to developed countries. The limit is financial and, even if surgery is offered at reduced cost, only few lucky patients will benefit from this kind of help.

The second is to perform surgical missions overseas in a selected local Institution, providing a full team covering all the medical and substantial needs. Usually, the visiting teams spend short periods, several times a year, for a variable number of years. This is the most widespread model, but it is expensive and the training “on-site” is usually ineffective and suboptimal (8).

The third way is to establish a long-term collaboration between a local hospital and a well-known experienced international partner, in order to create autonomous local cardiovascular services. This model needs long period of development, great financial investment, and very strong local will (5). It is the most difficult model to implement, but the one with the greatest potential for success in terms of autonomy and sustainability in the long run.

A very interesting survey published by Nguyen et al. (2) showed how several NGOs still play a crucial role in the CHD field, providing clinical services and infrastructure support to cardiovascular programs in developing countries. They identified 80 international NGOs supporting cardiovascular programs in 92 countries: the majority operated in South and Central America (42%) followed by Africa (18%), Europe (17%), Asia (17%), and Asia-Western Pacific (6%). From this analysis it was clear that between one mission and another only a few local centers worked independently, the others being completely dependent from international support (2). More than half (51%) of these NGOs supported between two and five missions per year performing surgery, catheter-based interventions, and education. Most of these NGOs had their headquarters in North America or Europe. The study highlights that the model of international cooperation through the NGOs is experiencing a progressive decline related to lack of funding, reduction of volunteers, and unreliability of local partners, who fail to have an independent activity alternative to the missions, and the complete lack of collaboration and network among the various NGOs (2).

One year ago, the WHO declared the Coronavirus disease 2019 (COVID-19) a pandemic and the world changed suddenly. The impact of the pandemic on the Italian cardiac surgery system and the consequent reorganization of the health model especially in the most affected areas as northern Italy have already been published by us (9, 10).

The effects of the pandemic on the health systems around the world were immediate and the consequent economic downturn, including the drastic reduction in traveling, has had a particularly negative impact on the activities of the different NGOs.

Our survey provides a partial figure of the situation: in 2019, 21 Italian congenital cardiac surgeons operated on 471 patients with CHD overseas and 251 in Italy, for a total of 722 children. In 2020, following the pandemic, the reduction in interventions was 95% for overseas and 86% for national activities, respectively. We do not know what happened to the more than 700 children who, based on the numbers of 2019, would have been operated in 2020 and in 2021.

We can only assume that, since the sickest children cannot wait for an operation, many of them are probably deceased. Therefore, the failure to treat those patients could be counted as deaths indirectly related to the pandemic.

The Italian survey confirmed that the most popular international cooperation system is weak, completely dependent on the activity of the NGOs from the rich part of the world, far away from the claimed goal of autonomy and sustainability (2, 6, 11, 12), and that the pandemic has dramatically exacerbated the situation.

A parallel result pointed out by this work is the lack of collaboration in sharing objectives and experiences between the various Italian centers: few know what other centers do and there is no structured organization that can coordinate and optimize efforts and resources.

What does this situation teach us? That we do need to think of a completely different operating model on a global level. International bodies or Scientific Societies such as the World Society for Pediatric and Congenital Heart Disease, the World Heart Foundation, and the World Health Organization must play a leadership role in coordination and organization. Few large projects with strong financial support in well-identified geographic areas are better than many small projects totally dependent on single NGOs or even single people.

The Hub & Spoke operating model (13, 14), which has shown its effectiveness even during emergency situations such as the pandemic in Italy (9, 10), could be adopted in the future to reduce the growing problem of CHD in developing countries.

The goal would be to identify for each continent a few centers (Hubs) with cardiac surgeons, cardiologists, anesthetists, intensivists, perfusionists, nurses, and well-trained administrative staff to which all other minor centers (Spoke) able of providing diagnostic evaluation, preventive cardiology, and outpatients monitoring can refer continuously.

Disparity and inadequacy of treatment of CHD remains an unsolved problem in developing countries and the pandemic has made this limitation particularly evident.

A global strategy is needed to improve cardiovascular care delivery in poor countries and, in this moment of greatest difficulty due to COVID disease, the maximum effort should be made in view of a better future. All the greatest historical revolutions, in fact, followed periods of great crisis, such as the end of the Middle Ages, from which the nations recovered with difficulty and profoundly transformed societies, ultimately leading to a rebirth, the Renaissance.

## Conclusions

One year ago, the WHO declared the coronavirus disease 2019 (COVID-19) a pandemic and the world changed suddenly. The pandemic had immediate and tremendous effects on health systems around the world and a particularly negative impact on the activities of the different NGOs involved in the treatment of patients with CHD. In Italy the reduction of this activity in 2020 was 96% for overseas and 86% for on-site charities. We should reconsider the actual international cooperation models in light of more efficient ways to increase autonomy and sustainability in developing countries.

## Data Availability Statement

The raw data supporting the conclusions of this article will be made available by the authors, without undue reservation.

## Author Contributions

AG, FC, AV, and AF contributed to conception and design of the study and wrote sections of the manuscript. CP, SM, NU, VV, FS, GL, GS, GG, BM, SF, GO, SA, and LG provided us with data on humanitarian activities carried out in Italy and overseas. All authors contributed to manuscript revision, read, and approved the submitted version.

## Conflict of Interest

The authors declare that the research was conducted in the absence of any commercial or financial relationships that could be construed as a potential conflict of interest.

## Publisher's Note

All claims expressed in this article are solely those of the authors and do not necessarily represent those of their affiliated organizations, or those of the publisher, the editors and the reviewers. Any product that may be evaluated in this article, or claim that may be made by its manufacturer, is not guaranteed or endorsed by the publisher.
