# Supplementary material for: Impact of COVID-19 Pandemic on the Italian Humanitarian Congenital Cardiac Surgery Activity: What No One Tells You
Source: Front Cardiovasc Med. 2021 Jul 28;8:705029. doi: 10.3389/fcvm.2021.705029 (PMC8355370; doi:10.3389/fcvm.2021.705029)
Supplement: Supplementary file 1 [file Data_Sheet_1.docx]

***QUESTIONNAIRE ON THE PANDEMIC COVID-19 IMPACT ON HUMANITARIAN PEDIATRIC CARDIOLOGY AND CARDIAC SURGERY***

1. Does your organization operate children with heart disease from developing countries?
2. Does your organization perform overseas surgical missions in developing countries?

*If YES:*

- 1. How many times in 2019?
  2. In which countries?

1. Are these surgical missions abroad carried out by your team or by different teams that you sponsor?
2. How many patients have been operated on abroad in this way in 2019?
3. Did the Covid-19 Pandemic reduce this activity in 2020?

*If YES:*

- 1. How much (percentage)?

1. In your activity, is it planned to operate in your country patients thank your financial support?

*If YES:*

- 1. Do you have any agreement with one hospital or more hospitals?

1. How many patients have been operated on in your country in this way in 2019?
2. Did the Covid-19 Pandemic reduce this activity in 2020?
   1. If YES: how much (percentage)?
3. When do you think you can start your activity again?
4. Any comments on the impact of the covid-19 pandemic on your activity and more generally on humanitarian medicine?
